# Surgery for acute cholecystitis in severely comorbid patients: a population-based study on acute cholecystitis

**DOI:** 10.1186/s12876-022-02453-0

**Published:** 2022-08-04

**Authors:** Erik Osterman, Louise Helenius, Christina Larsson, Sofia Jakobsson, Tamali Majumder, Anders Blomberg, Jennie Wickenberg, Fredrik Linder

**Affiliations:** 1 Department of Surgery, Gävle Hospital, 80187 Gävle, Gävleborg Region Sweden; 2 Department of Surgery, Uppsala University Hospital, Uppsala, Uppsala Region Sweden; 3 Department of Surgical Sciences, Uppsala University, Uppsala, Sweden; 4 Department of Immunology, Genetics and Pathology, Uppsala University, Uppsala, Sweden; 5 Centre for Research and Development, Gävle, Gävleborg Region Sweden

**Keywords:** Acute cholecystitis, Population-based, Retrospective, Survival, Treatment

## Abstract

**Background:**

International guidelines recommend emergency cholecystectomy for acute cholecystitis in patients who are healthy or have mild systemic disease (ASA1-2). Surgery is also an option for patients with severe systemic disease (ASA3) in clinical practice. The study aimed to investigate the risk of complications in ASA3 patients after surgery for acute cholecystitis.

**Method:**

1 634 patients treated for acute cholecystitis at three Swedish centres between 2017 and 2020 were included in the study. Data was gathered from electronic patient records and the Swedish registry for gallstone surgery, Gallriks. Logistic regression was used to assess the risk of complications adjusted for confounding factors: sex, age, BMI, Charlson comorbidity index, cholecystitis grade, smoking and time to surgery.

**Results:**

725 patients had emergency surgery for acute cholecystitis, 195 were ASA1, 375 ASA2, and 152 ASA3. Complications occurred in 9% of ASA1, 13% of ASA2, and 24% of ASA3 patients. There was no difference in 30-day mortality. ASA3 patients stayed on average 2 days longer after surgery. After adjusting for other factors, the risk of complications was 2.5 times higher in ASA3 patients than in ASA1 patients. The risk of complications after elective surgery was 5% for ASA1, 13% for ASA2 and 14% for ASA3 patients. Regardless of ASA 18% of patients treated non-operatively had a second gallstone complication within 3 months.

**Conclusion:**

Patients with severe systemic disease have an increased risk of complications but not death after emergency surgery. The risk is lower for elective procedures, but a substantial proportion will have new gallstone complications before elective surgery.

*Trial registration*: Not applicable.

**Supplementary Information:**

The online version contains supplementary material available at 10.1186/s12876-022-02453-0.

## Background

Acute cholecystitis is a common complication of cholecystolithiasis. Cholecystitis may also occur in critically ill patients due to the disruption of the biliary peristalsis, and in a minority for other reasons [1]. Laparoscopic cholecystectomy is the recommended treatment if possible. Severe systemic disease as measured by the American Society of Anesthesiologists classification (ASA ≥ 3) [2] or Charleston Comorbidities index (CCI ≥ 7) [3] is considered a contraindication for surgery in the most recent Tokyo guidelines [4]. The World Society of Emergency Surgery (WSES) guidelines give no firm recommendations but state that patients with ASA3 or aged above 80 are at high risk of morbidity and mortality [5]. Despite this, emergency cholecystectomy is the most common emergency general surgery procedure in the elderly [6, 7]. The experience from our centres is that patients with ASA 3 are routinely operated upon without a large increase in morbidity and mortality, but this has not been systematically evaluated.

Non-operatively managed (NOM) patients may be considered for surgery later to avoid new complications from cholecystolithiasis, e.g. another cholecystitis, pancreatitis, cholangitis, and gallstone ileus. Late cholecystectomy is considered worse than early cholecystectomy because of higher costs, risk of additional gallstone complications and worse quality of life [8–11]. How common it is for patients to have complications while waiting for surgery, or for patients not considered candidates for surgery to have another complication is debated and estimates range from 2.5 to 22% while waiting for surgery and the lifetime risk of new gallstone complications is estimated to be between 10 and 50%. [1, 12–14]

There is a registry in Sweden covering almost all the emergency and elective cholecystectomies performed since 2005, The Swedish Registry of Gallstone Surgery and Endoscopic Retrograde Cholangiopancreatography, Gallriks, from which regular reports about the quality of surgical treatment and research are published [15, 16]. No corresponding registry with detailed data exists for NOM patients in Sweden.

### Aim and hypotheses

The study aimed to investigate if it is safe to perform emergency cholecystectomies compared to NOM in patients deemed too sick to have surgery according to the Tokyo guidelines (ASA ≥ 3 and CCI ≥ 7). Additionally, we wanted to investigate the length of stay and outcomes after surgery compared to patients who are healthy or have mild systemic disease (ASA1-2 or CCI < 7).

The hypothesis was that ASA3 patients are potential candidates for surgery and have a similar rate of complications compared to patients with fewer comorbidities and that patients treated conservatively are at excessive risk for second events which motivates cholecystectomy even in comorbid patients.

## Method

The Swedish Ethical Review Authority approved the study, dnr 2021–00,862. The manuscript was prepared using the RECORD guidelines. [17]

### Data collection

Data for all patients with a diagnosis of acute cholecystitis and cholecystitis with and without gallstones (ICD K80.0, K80.1, K81.0-K801.9) between 2017 and 2020 was requested from the administrative data offices of Region Gävleborg and Region Uppsala. Electronic patient records (EPR) were then screened, and data was recorded by five of the authors after an introduction to the tools and variables. Patients with acute cholecystitis based on Tokyo guidelines diagnostic criteria and no other diagnosis explaining the symptoms, e.g. cholangitis or pancreatitis were included in the study. [18]

Study data were collected and managed using REDCap electronic data capture tools hosted at Uppsala University [19, 20]. Additional data for patients who had a cholecystectomy was then requested from Gallriks using personal identification numbers to supplement the EPR data [15]. For the final data set, missing ASA classification from EPR was supplemented from Gallriks and faulty dates were checked against the EPR and Gallriks and corrected.

Patients were stratified according to the treatment choice at index cholecystitis (surgery or NOM, including cholecystostomy).

### Variables

The primary outcome for patients treated with surgery was peri- and postoperative complications which were classified as: no complication, complications treated without general anaesthesia (Clavien-Dindo ≤ 3a), and complications requiring new surgery, leading to organ failure, ICU care or death (Clavien-Dindo ≥ 3b). [21]

For NOM patients the primary outcome was second gallstone complication or index cholecystitis treatment failure, i.e. recurrence, and the date of complication or diagnosis of treatment failure.

Data on second gallstone complications and handling of the gallbladder (surgery or NOM)

was collected from the EPR with follow-up set to the date of EPR review for time-to-event analysis. Date of death or follow-up without death was collected from EPR for all patients and from Gallriks for patients who had surgery. Time to second gallstone complication was defined as the time from diagnosis to new gallstone disease with censoring for follow-up and death. The secondary outcome, length of stay, was collected from the EPR.

The exposure, ASA classification, was determined from EPR with reference to examples translated to Swedish. [2, 22]

Sex, age, smoking, BMI, CCI and separate comorbidities, cholecystitis grade and time from symptoms to surgery were included as potential confounders. Comorbidities were recorded as cardiovascular disease (heart disease, peripheral vascular and cerebrovascular disease), diabetes, pulmonary disease (COPD, asthma, or other chronic pulmonary diseases), other diseases (grouped due to the small number of cases, dementia, kidney failure, liver failure, tumours) and increased bleeding risk (anticoagulant use or hereditary bleeding disorders) using data from EPR and Gallriks. The cholecystitis grade was classified as Grade 1 or grade 2–3 since complete data on organ and systemic dysfunction was not readily available in the electronic patient records, patients without grade II features were assumed to not have organ dysfunction. [18]

### Statistics

Patients were stratified by index treatment: surgery or NOM, and ASA classification to analyse outcomes and demographics. Differences between groups were tested with the Chi-Square test for categorical variables and Mann–Whitney U and Kruskal–Wallis test for discrete variables to avoid normality assumptions.

Gallriks data was compared with the data recorded in the database regarding complications with the Cohen’s kappa coefficient and Pearson’s correlation coefficient for continuous data.

Logistic regressions were used to investigate the risk of complications. Sex, age, smoking, BMI, CCI, diabetes, cardiovascular disease, pulmonary disease, other comorbidities, grade of cholecystitis, and time to surgery were analysed individually to investigate the correlation with postoperative outcomes. The adjusted analysis excluded the individual comorbidities since these are included in ASA and CCI measurements.

The risk of readmission and 30-day mortality and the length of stay for ASA3 patients treated with and without surgery was investigated with propensity score matching. Patients were matched on age, sex, CCI, BMI, grade of cholecystitis, centre, and time from symptoms to admission. The MatchIt R-package was used, with the method “nearest”, caliper of 0.1 and a generalised linear model to assess the distance [23]. Differences in mortality and readmission were tested with logistic regression and length of stay was tested with linear regression. Standard errors were calculated using a cluster robust method.

Sensitivity analysis on the proportion of complications in different ASA classifications was performed using data from Gallriks.

Cox regression was used to investigate time to second gallstone complication in NOM patients, censoring was used.

Statistics were calculated with R version 3.14 (Vienna, Austria). Cases with missing data were included for analysis in differences between groups and reported in tables while they were removed in regression analysis. P-values of < 0.05 were considered statistically significant, analysis was exploratory without correction for multiple testing.

## Results

1 634 patients were included after the exclusion of 245 patients (6 minors, 3 declining studies, 232 without acute cholecystitis and 4 with cholecystitis before 2017), Fig. 1.

**Fig. 1:**
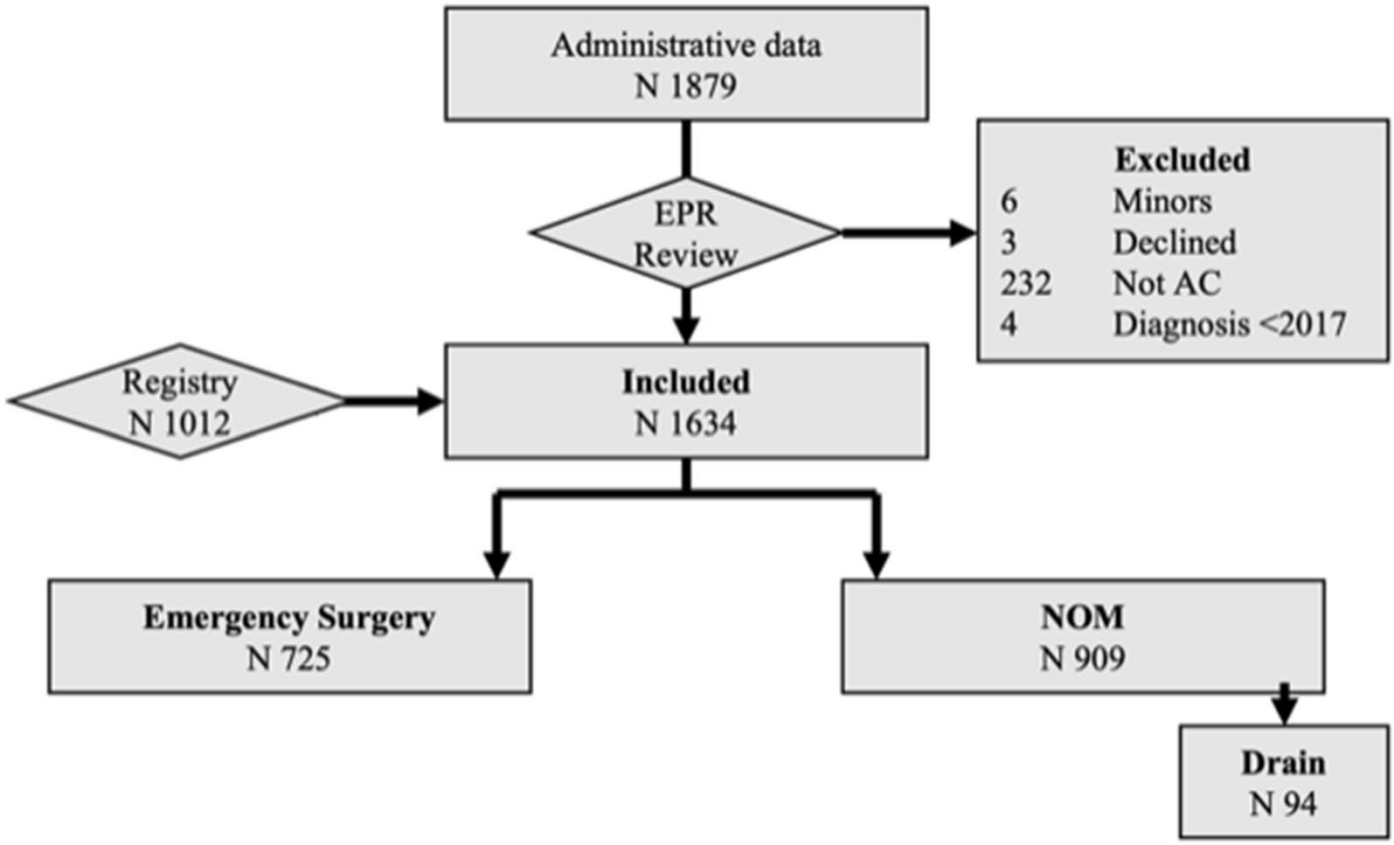
Patient flow chart. NOM – non-operative management

### Demography

Some 777 out of 1 634 patients (48%) were female. The mean age was 64 years, standard deviation (SD) was 17. Seven hundred twenty-five (44%) had surgery, 815 (50%) were treated with best supportive care ± antibiotics, and 94 (6%) received a cholecystostomy. Cholecystostomy treatment was attempted in 3 ASA3 patients who later had surgery, these are included in the surgery group. Patient characteristics are described in Table 1. ASA-classification and CCI strongly correlated, Additional file 1: Fig. S1.

**Table 1:** Demographics and clinical data by treatment strategy at index cholecystitis

		Total	Surgery	NOM		*p *
				Conservative	Drain	
Total	Total	1 634 (100%)	725 (44%)	815 (50%)	94 (6%)	–
Sex	Male	857 (52%)	373 (51%)	426 (52%)	58 (62%)	0.17
	Female	777 (48%)	352 (49%)	389 (48%)	36 (38%)	
Age	Mean (SD)	64.2 (± 17.1)	57.4 (± 15.1)	69.0 (± 17.0)	76.0 (± 11.3)	< 0.001
BMI	Mean (SD)	28.5 (± 5.5)	28.9 (± 5.1)	28.3 (± 5.9)	27.0 (± 5.7)	0.003
	Missing	117 (7.2%)	39 (5.4%)	71 (8.7%)	7 (7.4%)	
Smoking	No/previous	1 486 (91%)	661 (91%)	740 (91%)	85 (90%)	0.93
	Yes, current	148 (9%)	64 (9%)	75 (9%)	9 (10%)	
Year of diagnosis	2017	401 (25%)	157 (22%)	218 (27%)	26 (28%)	0.057
	2018	405 (25%)	171 (24%)	205 (25%)	29 (31%)	
	2019	394 (24%)	194 (27%)	180 (22%)	20 (21%)	
	2020	434 (27%)	203 (28%)	212 (26%)	19 (20%)	
ASA	1	300 (18%)	195 (27%)	100 (12%)	5 (5%)	< 0.001
	2	731 (45%)	375 (52%)	328 (40%)	28 (30%)	
	3	515 (32%)	152 (21%)	316 (39%)	47 (50%)	
	4	87 (5%)	2 (0%)	71 (9%)	14 (15%)	
	5	1 (0%)	1 (0%)	0 (0%)	0 (0%)	
CCI	Mean (SD)	3.2 (± 2.6)	2.0 (± 1.9)	4.0 (± 2.8)	5.4 (± 2.7)	< 0.001
Cholecystitis Grade	G1	798 (49%)	425 (59%)	338 (41%)	35 (37%)	< 0.001
	G2-3	729 (45%)	268 (37%)	402 (49%)	59 (63%)	
	Missing	107 (7%)	32 (4%)	75 (9%)	0 (0%)	
Length of stay	Mean (SD)	4.7 (± 5.6)	4.1 (± 5.0)	4.6 (± 5.8)	9.4 (± 5.3)	< 0.001
	Missing	52 (3.2%)	0 (0%)	52 (6.4%)	0 (0%)	
Readmitted within 30 days	No	1 445 (88%)	681 (94%)	692 (85%)	72 (77%)	< 0.001
	Yes	189 (12%)	44 (6%)	123 (15%)	22 (23%)	
30d Mortality	Alive	1,481 (91%)	708 (98%)	694 (85%)	79 (84%)	< 0.001
	Dead	34 (2%)	2 (0%)	25 (3%)	7 (7%)	
	Missing	119 (7%)	15 (2%)	96 (12%)	8 (9%)	
1y mortality	Alive	1,426 (87%)	704 (97%)	653 (80%)	69 (73%)	< 0.001
	Dead	89 (5%)	6 (1%)	66 (8%)	17 (18%)	
	Missing	119 (7%)	15 (2%)	96 (12%)	8 (9%)	

Fisher Exact Test used for categorical variables. Kruskal-Wallis test for continuous variables

*ASA* American Society of Anesthesiologists physical status classification, *BMI* Body Mass Index, *CCI* Charlson Comorbidity Index, *NOM* Non-operative management

The proportion of patients having surgery increased between 2017 and 2019 and was stable for 2020 despite the initial waves of the COVID-19 pandemic. There was no ASA-dependent difference in time from the debut of symptoms to diagnosis. Length of stay was longer in admitted NOM patients and even longer if they had a cholecystostomy, however, 52 NOM patients were not admitted (denoted as missing in Table 1).

### Completeness of registry data

Of the 725 patients who had an emergency cholecystectomy, Gallriks data was available for 658 (91%). Gallriks data was available for 258 of 282 (94%) patients who had elective surgery after their cholecystitis. Similar coverage was seen for patients with emergency (67 of 72, 93%) and elective surgery (27 of 30) after the first new gallstone complication. Two patients had surgery after a third gallstone complication.

The concordance for categorical variables in EPR and Gallriks for patients having emergency surgery are presented in Additional file 1: Table S1. BMI had a correlation coefficient of 0.96 in 776 patients with reported BMI in both databases. Additional file 1: Table S2 details the differences between the EPR and registry ASA classification.

### Comorbidities and complications

Demographics and follow-up data stratified by ASA classification are presented in Table 2 for patients who had emergency surgery. One patient with ASA 5 was merged with ASA 4. Overall there were 104 complications in 725 (14%) patients having emergency cholecystectomies. There was a strong correlation between ASA classification and complications and slightly more patients with ASA3 had open surgery, either planned or due to conversion from laparoscopy. Fourteen of 181 (8%) patients with CCI ≥ 7 had surgery in the emergency setting, there were 5 complications (38%). There was no comorbidity dependent difference in time to surgery from being admitted. The distribution of comorbidities by ASA is detailed in Additional file 1: Table S3.

**Table 2:** Demographics and outcomes for patients where emergency surgery was performed by comorbidity level

		Total	ASA1	ASA 2	ASA 3	ASA4-5	*p*
Total	Total	725 (100%)	195 (27%)	375 (52%)	152 (21%)	3 (0%)	
Sex	Male	373 (51%)	102 (52%)	189 (50%)	80 (53%)	2 (67%)	0.91
	Female	352 (49%)	93 (48%)	186 (50%)	72 (47%)	1 (33%)	
Age	Mean (SD)	57.4 (± 15.1)	48.7 (± 13.0)	58.7 (± 14.7)	65.1 (± 13.3)	62.2 (± 10.6)	< 0.001
BMI	Mean (SD)	28.9 (± 5.1)	27.3 (± 4.0)	29.0 (± 4.5)	30.6 (± 6.9)	29.1 (± 5.2)	< 0.001
	Missing	39 (5.4%)	16 (8.2%)	22 (5.9%)	1 (0.7%)	0 (0%)	
CCI	Mean (SD)	2.0 (± 1.9)	0.8 (± 1.0)	2.0 (± 1.5)	3.7 (± 2.2)	4.7 (± 3.2)	< 0.001
Cholecystitis Grade	G1	425 (59%)	107 (55%)	218 (58%)	98 (64%)	2 (67%)	0.54
	G2-3	268 (37%)	74 (38%)	143 (38%)	50 (33%)	1 (33%)	
	Missing	32 (4%)	14 (7%)	14 (4%)	4 (3%)	0 (0%)	
Length of stay	Mean (SD)	4.1 (± 5.0)	3.1 (± 2.2)	3.6 (± 2.6)	6.3 (± 8.8)	19.7 (± 28.0)	< 0.001
Surgical approach	Laparoscopic	560 (77%)	152 (78%)	298 (79%)	108 (71%)	2 (67%)	0.038
	Open	42 (6%)	12 (6%)	14 (4%)	15 (10%)	1 (33%)	
	Converted	58 (8%)	12 (6%)	31 (8%)	15 (10%)	0 (0%)	
	Missing	65 (9%)	19 (10%)	32 (9%)	14 (9%)	0 (0%)	
Operative Complication	No	621 (86%)	177 (91%)	327 (87%)	115 (76%)	2 (67%)	< 0.001
	Clavien 1-3a	76 (10%)	16 (8%)	38 (10%)	22 (14%)	0 (0%)	
	Clavien 3b +	28 (4%)	2 (1%)	10 (3%)	15 (10%)	1 (33%)	
Stay after surgery	Mean (SD)	3 (± 5)	2 (± 2)	2 (± 2)	4 (± 8)	18 (± 28)	< 0.001
	Missing	13 (2%)	6 (3%)	6 (2%)	1 (1%)	0 (0%)	
Readmitted within 30 days	No	681 (94%)	186 (95%)	352 (94%)	140 (92%)	3 (100%)	0.54
	Yes	44 (6%)	9 (5%)	23 (6%)	12 (8%)	0 (0%)	
30d mortality	Alive	708 (98%)	188 (96%)	370 (99%)	147 (97%)	3 (100%)	0.052
	Dead	2 (0%)	0 (0%)	0 (0%)	2 (1%)	0 (0%)	
	Missing	15 (2%)	7 (4%)	5 (1%)	3 (2%)	0 (0%)	
1y mortality	Alive	704 (97%)	188 (96%)	369 (98%)	144 (95%)	3 (100%)	0.007
	Dead	6 (1%)	0 (0%)	1 (0%)	5 (3%)	0 (0%)	
	Missing	15 (2%)	7 (4%)	5 (1%)	3 (2%)	0 (0%)	

Fisher Exact Test used for categorical variables. Kruskal-Wallis test for continuous variables

*ASA* American Society of Anesthesiologists physical status classification, *BMI* Body Mass Index, *CCI* Charlson Comorbidity Index

Univariable and multivariable logistic regression was calculated with any peri- and postoperative complications as the outcome, Table 3. An increased risk of perioperative complications was seen for increasing ASA, CCI score and age. ASA correlated with complications in multivariable analysis, the odds ratio (OR) of any complication was 1.4 and 2.5 for ASA 2 and ASA 3 respectively. When the analysis was limited to severe complications, the OR was 4.1 (non-significant) and 13.2 (p 0.020) in ASA2 and ASA3 patients. The average stay after surgery if there were complications was 6.5 (SD 11) days compared to 1.9 (SD 1.8) days if not (*p* < 0.001).

**Table 3:** Demographics and outcomes for NOM patients by comorbidity level

		Total	ASA1	ASA 2	ASA 3	ASA4-5	*p*
Total	Total	909 (100%)	105 (12%)	356 (39%)	363 (40%)	85 (9%)	
Sex	Male	484 (53%)	46 (44%)	179 (50%)	202 (56%)	57 (67%)	0.006
	Female	425 (47%)	59 (56%)	177 (50%)	161 (44%)	28 (33%)	
Age	Mean (SD)	69.7 (± 16.6)	51.5 (± 15.6)	67.4 (± 16.5)	75.5 (± 13.0)	76.9 (± 13.1)	< 0.001
BMI	Mean (SD)	28.2 (± 5.8)	28.2 (± 5.5)	28.0 (± 4.7)	28.5 (± 6.7)	27.3 (± 6.7)	0.36
	Missing	78 (8.6%)	15 (14.3%)	28 (7.9%)	29 (8.0%)	6 (7.1%)	
CCI	Mean (SD)	4.1 (± 2.8)	1.0 (± 1.2)	2.9 (± 1.9)	5.3 (± 2.3)	7.8 (± 2.9)	< 0.001
Cholecystitis Grade	G1	373 (41%)	27 (26%)	125 (35%)	180 (50%)	41 (48%)	< 0.001
	G2-3	461 (51%)	54 (51%)	199 (56%)	166 (46%)	42 (49%)	
	Missing	75 (8%)	24 (23%)	32 (9%)	17 (5%)	2 (2%)	
Drain complication	No	60 (7%)	5 (5%)	21 (6%)	26 (7%)	8 (9%)	0.13
	Yes	33 (4%)	0 (0%)	7 (2%)	20 (6%)	6 (7%)	
	Missing	816 (90%)	100 (95%)	328 (92%)	317 (87%)	71 (84%)	
Length of stay	Mean (SD)	5.2 (± 6.0)	3.6 (± 9.3)	4.4 (± 6.1)	5.8 (± 4.6)	7.4 (± 5.1)	< 0.001
	Missing	52 (5.7%)	19 (18.1%)	21 (5.9%)	12 (3.3%)	0 (0%)	
Readmitted within 30 days	No	764 (84%)	95 (90%)	306 (86%)	300 (83%)	63 (74%)	0.014
	Yes	145 (16%)	10 (10%)	50 (14%)	63 (17%)	22 (26%)	
New gall stone complication	No complication	614 (68%)	85 (81%)	245 (69%)	229 (63%)	55 (65%)	0.007
	Complication	265 (29%)	18 (17%)	101 (28%)	122 (34%)	24 (28%)	
	Missing	30 (3%)	2 (2%)	10 (3%)	12 (3%)	6 (7%)	
30d mortality	Alive	773 (85%)	90 (86%)	321 (90%)	296 (82%)	66 (78%)	< 0.001
	Dead	32 (4%)	0 (0%)	4 (1%)	18 (5%)	10 (12%)	
	Missing	104 (11%)	15 (14%)	31 (9%)	49 (13%)	9 (11%)	
1y mortality	Alive	722 (79%)	88 (84%)	314 (88%)	271 (75%)	49 (58%)	< 0.001
	Dead	83 (9%)	2 (2%)	11 (3%)	43 (12%)	27 (32%)	
	Missing	104 (11%)	15 (14%)	31 (9%)	49 (13%)	9 (11%)	

Fisher exact test used for categorical variables. Kruskal-wallis test for continuous variables

*ASA* American Society of Anesthesiologists physical status classification, *BMI* Body Mass Index, *CCI* Charlson Comorbidity Index

Complications were less common in patients that had elective surgery after their first cholecystitis, with 33 of 282 (12%) having any complication of which 13 had a severe complication. Three of 56 (5%) ASA1 patients, 21 of 161 (13%) ASA2, and 9 of 63 (14%) ASA3 patients had complications after elective cholecystectomy.

Seventy-two patients had emergency surgery for recurrent disease, 19 of which had peri/postoperative complications (26%). Thirty patients had elective surgery after recurrence, of which four had complications.

In sensitivity analysis complications at/after emergency surgery were correlated with the ASA classification when registry ASA or registry complications were used, but not both, see Additional file 1: Table S4. Age > 65 was associated with complications but when analysing only patients > 65 years old no statistically significant correlation between ASA and complications was seen nor when age groups were compared within each ASA group, Additional file 1: Table S5.

### Risk of second complications from gallstones and treatment failure

**Fig. 2:**
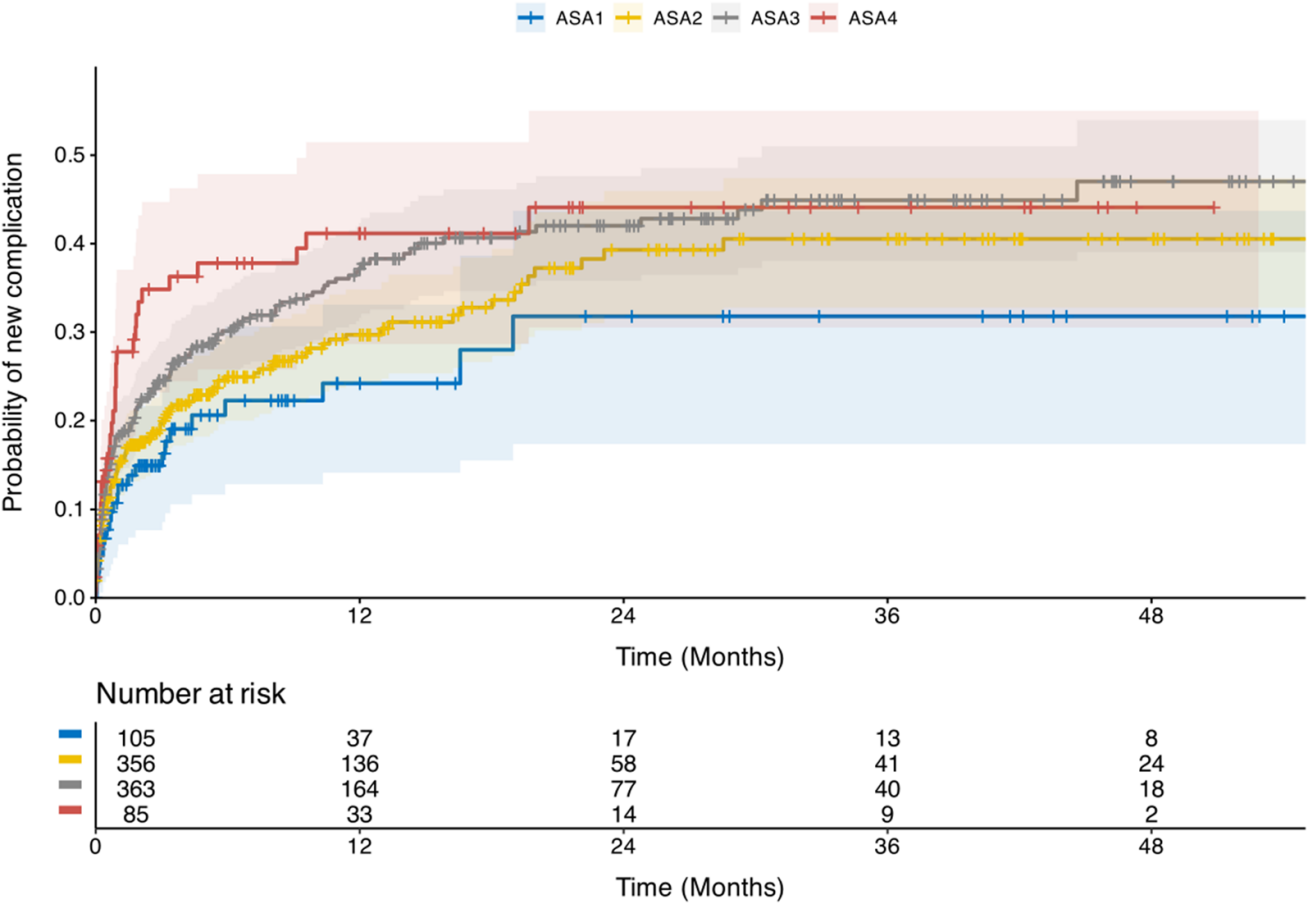
Time to treatment failure or second gallstone complication in non-operatively managed patients by comorbidity status, ASA: American Society of Anesthesiologists

Demographics and follow-up data for NOM patients are presented in Table 4. NOM patients had a high risk of treatment failure or second complication from gallstones (excluding pain), i.e. recurrence of complications from gallstones. Of the 909 NOM patients, 145 (16%) were readmitted within 30 days and 265 had a recurrence during the study period with an average follow-up of 14 months before censoring, death, surgery, or recurrence. Two hundred twenty-nine of 265 (86%) patients with a recurrence were admitted for an average of 6.1 days (SD 6.4). Of the 145 patients readmitted within 30 days, 51 had an early recurrence, possibly treatment failure, in addition, 13 patients had an early recurrence and were not admitted. In total, 64 of 778, (8%) recurred within 1 month. At 3 months 129 patients (18%) had recurred, at 1 year 213 (33%) and at 2 years 239 (40%). The risk of new complications and readmission within 30 days was higher in patients with ASA3-5, Table 2 and Fig. 2.

**Table 4:** Logistic regression for risk of postoperative complications by comorbidities. Multivariable regression for the risk of postoperative complications

	Unadjusted		Adjusted	
Factor (reference)	OR (95% CI)	*p*	OR (95% CI)	*p*
Female sex (Male)	0.89 (0.59–1.36)	0.597	0.86 (0.55–1.35)	0.525
Age (continuous)	1.02 (1.01–1.04)	0.004	0.99 (0.97–1.02)	0.647
BMI (continuous)	0.98 (0.94–1.02)	0.390	0.97 (0.93–1.02)	0.249
ASA 2 (ASA1)	1.44 (0.82–2.56)	0.208	1.42 (0.73–2.75)	0.300
ASA3	3.16 (1.72–5.82)	< 0.001	2.51 (1.11–5.68)	0.027
ASA 4	0.01 (0–2 × 10^17^)	0.842	0.01 (0–1 × 10^17^)	0.833
CCI (continuous)	1.24 (1.12–1.38)	< 0.001	1.16 (0.94–1.42)	0.164
Vascular disease (No)	2.08 (1.34–3.23)	0.001		
Diabetes (No)	1.14 (0.59–2.19)	0.704		
Pulmonary disease (No)	1.03 (0.54–1.97)	0.937		
Other comorbidities (No)	2.38 (1.29–4.41)	0.006		
Bleeding risk (No)	2.44 (1.17–5.10)	0.018		
Cholecystitis grade 2–3 (Grade 1)	1.39 (0.91–2.13)	0.126	1.47 (0.88–2.45)	0.140
Current smoker (Not smoker)	0.71 (0.32–1.61)	0.417	0.75 (0.33–1.73)	0.502
Time from symptoms to surgery (continuous)	1.03 (0.97–1.11)	0.321	1 (0.93–1.09)	0.910

Degrees of freedom were 1 for all unadjusted tests except ASA-classification where it was 3. Degrees of freedom for adjusted test were 9

*ASA* American Society of Anesthesiologists physical status classification, *BMI* Body Mass Index, *CCI* Charlson Comorbidity Index, *OR* Odds Ratio, Vascular disease: Includes cardiac, cerebral, and peripheral vascular disease

95% CI: 95% Confidence interval

### Outcomes in ASA 3 patients dependent on treatment choice

After matching patients operated with NOM patients there were 116 ASA 3 patients in each group. The groups were not completely balanced on sex, centre and grade as shown in Additional file 1: Fig. S2. There was no difference in 30-day mortality (p 0.41) or 30-day readmissions (*p* 0.14). The mean length of stay was 7 days for patients treated with surgery compared to 4 days for NOM patients (p 0.015).

## Discussion

### Risk vs benefit

In this population-based study, the risk of any peri/postoperative complication in ASA3 patients undergoing emergency surgery for acute cholecystitis was 24% compared to 9% and 13% in ASA1 and ASA2, respectively. This correlation was independent of sex, age, BMI, CCI, grade of cholecystitis, smoking and time to surgery and the risk of severe complications for ASA3 patients was 13-fold that of ASA1 patients. Even though there was an increased risk of severe complications in ASA3 patients there was no statistically significant difference in mortality at 30 days or 30-day readmissions. At 1 year, most of the mortality difference may be due to other diseases and not to surgery for cholecystitis. When comparing matched patients, the only difference was the length of stay during the index cholecystitis.

Seventeen per cent of NOM patients were readmitted for treatment failure or a second complication from gallstones within 1 month. The proportion of recurrences was 18% at 3 months and then slowly increased for the next two years before tapering off. At least 50% of recurrences could have been avoided with elective surgery within 3 months, with a lower risk of peri/postoperative complications for ASA3 patients compared to emergency surgery.

The absolute difference in risk of peri/postoperative complications between emergency and elective surgery for ASA1 and ASA2 patients was small, 0–4%. For ASA3 patients the difference was 10%, resulting in 40% less risk if operated upon in the elective setting. The acute systemic inflammation in ASA3 patients may make them more susceptible to pulmonary and cardiac events compared to ASA1-2 patients. It is somewhat surprising that 39% of ASA1-2 patients did not have later surgery even though there are strong recommendations regarding this. It is less surprising that 72% of ASA3 patients NOM did not have surgery later either.

The ASA3 patients who underwent emergency surgery were most likely selected to be the healthiest of the group, even though a few patients with more complicated cholecystitis may be included. NOM ASA3 patients had a higher proportion of both 30-day and 1-year mortality. In addition, CCI for ASA3 patients treated with surgery was lower than for those NOM (3.7 vs 5.3) and they were also 10 years younger on average (which accounts for one point of the CCI score). ASA3 patients did not wait longer for diagnosis or surgery which otherwise may have indicated that patients were operated upon because symptoms did not resolve on NOM.

### Previous evidence

The risk of complications after emergency cholecystectomy in previous Gallriks materials has been around 16% where most complications are postoperative rather than intraoperative [15, 24]. The proportion of cases reported to the registry was better than in a 2014 validation [16] which may lead to better coverage of complications even without the EPR data [25]. Despite this we failed to replicate the results using only information from the registry which may rather be an effect of misclassified ASA in the registry. Mehta et al. found that the risk of complications was 14% for emergency cholecystectomies in patients above 65 years old and that comorbidities correlated with complications and 30-day readmissions [6]. In octogenarians there is both a high risk of morbidity and mortality associated with emergency cholecystectomy [26], however NOM is associated with both repeat readmission and worse overall survival in the elderly [13]. Endo et al. found that CCI ≥ 6 was the point where mortality increased for patients who underwent emergency or planned cholecystectomy in a Japanese material, while no difference in complications between grade I and II cholecystitis was seen [27]. In the present study there were few patients with CCI scores above 6 who underwent surgery. The risk of new gallstone complications in NOM patients was higher than presented in a 2017 meta-analysis, but in line with some of the studies included in the meta-analysis and studies cited in the Tokyo and WSES guidelines at 40%. [1, 5, 12, 13]

### Limitations

Patients who were cared for by general practitioners were not included, and thus some patients may be missing from the entire population. Retrospective chart review and registry studies are limited to what is reported, and some information is bound to be missing. In addition, reporting may differ between rapporteurs. Efforts were undertaken to ensure that the interpretation and reporting of variables to the database was uniform, but it was not feasible to use dual data entry. Survival data was missing for 104 NOM patients with the distribution of missing data equal by ASA classification. These patients were assumed to be alive in the reporting of survival, thus the mortality may be higher. They were treated as missing for time to recurrence analyses which may bias these in the direction of too many recurrences but not readmissions since this was recorded separately. The percentage of recurrences, not considering censoring and surgery, was 29%, We did not separate calculous and acalculous cholecystitis in this study because patients who are operable benefit from surgery for acute acalculous cholecystitis and it is rare outside of otherwise critically ill patients who would not tolerate surgery.

### Strengths

Data from the registry is entered prospectively after surgery. Data on surgery was mostly complete and the direction of missingness is most likely in favour of missed complications rather than overreporting. We could not completely grade the severity of the disease since we lacked information about organ failure criteria, however, the proportion of patients with grade 1 cholecystitis was similar between groups, telling that this does not explain the difference in outcomes and the proportion of grade III cholecystitis is usually small and more likely treated with drainage [28]. We aimed to cover a modern material of all patients that could be considered candidates for surgery and to study the acute cholecystitis population seeking specialist care, we believe that this has been achieved in the current study. The populations were included from three centres representing two health care regions and different settings (community hospital, primary referral centre and university hospital). The individual variables from the CCI score were used to compare with the registry, and some diseases are not included in CCI e.g. hypertension and atrial fibrillation that would have been included in the registry definition of cardiovascular disease. Subsequently, the concordance for comorbidities reported in the registry and EPR was low except for diabetes which is well defined as a diagnosis and often noted in patient records. When analysing the risk of complications both data sources were used to capture as many complications as possible.

### Identifying those at risk

There is an increased risk of complications with emergency surgery, but the risk of failed NOM and recurrent gallstone disease makes emergency surgery an attractive alternative for low-risk surgical patients. For ASA3 patients it is important to clearly outline the alternatives and discuss management options with the patient. A majority will not relapse but those that do, do so early. The risk of complications seems to be the same at the second complication and 50% of recurrences can be avoided with timely elective surgery.

Future studies will aim to identify a subset of patients who have a low risk of complications even though they are technically ASA3, e.g. patients with only morbid obesity, myocardial infarctions many years in the past without functional limitations and TIA but no stroke. In addition, identification of the patients who will relapse could further aid the decision-making process. There are models to predict the risk of recurrent gallstone disease to target surgery at the population at most risk [29]. However, the risk is mostly determined by the type of gallstone complications where pancreatitis offers the highest risk while cholecystitis is the second worst. [29]

## Conclusion

There is an increased risk of peri/postoperative complications in ASA3 patients. There was no corresponding increase in 30-day readmissions or mortality. The risk of readmission and new gallstone complications was high in all patients treated non-operatively and correlated with increasing ASA classification. A discussion is necessary with patients who are potential candidates for surgery where the risks and benefits are explained clearly. If not candidates in the emergency setting, ASA3 patients could be prioritized for planned procedures due to the high risk of second gallstone complications while ASA1 and ASA2 patients are operated upon in the emergency setting.

## Supplementary Information

**Additional file1: Table S1** Concordance between EPR and Gallriks data for patients who had surgery. **Table S2** ASA as reported in Gallriks and assessed from EPR. **Table S3** Frequency of comorbidities by ASA in patients who had emergency surgery. **Table S4** Sensitivity analysis using registry ASA and complications. **Table S5 **ASA classification, Age and complications. **Fig S1** Boxplot of CCI score by ASA-classification. Only one patient was ASA5 and had a CCI score of 7, ASA: American Society of Anesthesiologists, CCI: Charlson Comorbidity Index. **Fig S2** Distance between unmatched and matched ASA3 patients.

## Data Availability

The data are not publicly available due to information that could compromise the privacy of research participants. The registry data supporting this study's findings are available from Gallriks (https://www.ucr.uu.se/gallriks/). Restrictions apply to the availability of these data why the authors cannot share them. EPR data are however available from the corresponding author upon reasonable request.

## Abbreviations

ASA: American Society of Anesthesiologists performance classification
BMI: Body mass index
CCI: Charlson comorbidity index
EPR: Electronic patient records
Gallriks: The swedish registry of gallstone surgery and endoscopic retrograde cholangiopancreatography
OR: Odds ratio
NOM: Non-operative management
*P*: *P*-Value
SD: Standard deviation
WSES: World Society of Emergency Surgery
1y: 1 Year
30d: 30 Days
95% CI: 95% Confidence intervals
