# Supplementary material for: Surgery for acute cholecystitis in severely comorbid patients: a population-based study on acute cholecystitis
Source: BMC Gastroenterol. 2022 Aug 4;22:371. doi: 10.1186/s12876-022-02453-0 (PMC9354429; doi:10.1186/s12876-022-02453-0)
Supplement: Supplementary file 1 — Additional file1: Table S1 Concordance between EPR and Gallriks data for patients who had surgery. Table S2 ASA as reported in Gallriks and assessed from EPR. Table S3 Frequency of comorbidities by ASA in patients who had emergency surgery. Table S4 Sensitivity analysis using registry ASA and complications. Table S5 ASA classification, Age and complications. Fig S1 Boxplot of CCI score by ASA-classification. Only one patient was ASA5 and had a CCI score of 7, ASA: American Society of Anesthesiologists, CCI: Charlson Comorbidity Index. Fig S2 Distance between unmatched and matched ASA3 patients. [file 12876_2022_2453_MOESM1_ESM.pdf]

Supplementary material

Surgery for acute cholecystitis in severely comorbid patients: A population-based study on acute cholecystitis

Supplementary Table 1: Concordance between EPR and Gallriks data for patients who had surgery

Variable	EPR (yes)	Gallriks (yes)	Concordance	p
**Current smoker**	57	25	0.49	<0.001
**ASA classification (1/2/3/4-5)**	195/375/152/2/1	216/357/94/1/0	0.39	<0.001
**Cardiovascular disease**	85	150	0.41	<0.001
**Diabetes**	85	76	0.88	<0.001
**Pulmonary disease**	68	51	0.62	<0.001
**Date of surgery**			0.95	<0.001
**Postoperative complications**	95	60	0.23	<0.001
**Accidental rupture of gallbladder**	161	266	0.31	<0.001

p-value for differences between EPR and registry
EPR: Electronic Patient Records
ASA: American Society of Anesthesiologists classification

Supplementary Table 2: ASA as reported in Gallriks and assessed from EPR

**ASA from Gallriks**	**ASA from EPR**				
	1	***2***	***3***	***4***	***5***
1	127	81	7	1	0
2	44	230	73	0	0
3	4	30	58	1	1
4	0	1	0	0	0

ASA: American Society of Anesthesiologists classification

Supplementary Table 3: Frequency of comorbidities by ASA in patients who had emergency surgery.

		Total	ASA1	ASA 2	ASA 3	ASA4-5	p
**CVD**	No	547 (75%)	190 (97%)	297 (79%)	58 (38%)	2 (67%)	< 0.001
	Yes	178 (25%)	5 (3%)	78 (21%)	94 (62%)	1 (33%)	
**Diabetes**	No	649 (90%)	195 (100%)	333 (89%)	118 (78%)	3 (100%)	< 0.001
	Yes	76 (10%)	0 (0%)	42 (11%)	34 (22%)	0 (0%)	
**Pulmonary disease**	No	643 (89%)	192 (98%)	336 (90%)	112 (74%)	3 (100%)	< 0.001
	Yes	82 (11%)	3 (2%)	39 (10%)	40 (26%)	0 (0%)	
**Other**	No	665 (92%)	195 (100%)	346 (92%)	123 (81%)	1 (33%)	< 0.001
	Yes	60 (8%)	0 (0%)	29 (8%)	29 (19%)	2 (67%)	
**Bleeding risk**	No	615 (85%)	173 (89%)	322 (86%)	117 (77%)	3 (100%)	< 0.001
	Yes	38 (5%)	0 (0%)	18 (5%)	20 (13%)	0 (0%)	
	NA	72 (10%)	22 (11%)	35 (9%)	15 (10%)	0 (0%)	

Comorbidities were recorded as cardiovascular disease (heart disease, peripheral vascular and cerebrovascular disease), diabetes, pulmonary disease (COPD, asthma, or other chronic pulmonary diseases), other diseases (grouped due to the small number of cases, dementia, kidney failure, liver failure, tumours) and increased bleeding risk (anticoagulant use or hereditary bleeding disorders). Bleeding risk was assessed from Gallriks data, hence the missing data is for patients missing from Gallriks.
ASA: American Society of Anesthesiologists classification

Supplementary Table 4: Sensitivity analysis using registry ASA and complications

	Registry ASA			Registry complications			Registry ASA and complications		
**ASA**	**Total**	**C**	**%**	**Total**	**N**	**%**	**Total**	**C**	**%**
**1**	216	26	12%	195	8	4%	216	12	6%
**2**	347	50	14%	375	28	7%	347	31	9%
**3**	94	24	26%	152	19	13%	94	12	13%

ASA: American Society of Anesthesiologists
C: Peri/postoperative complications

Supplementary Table 5: ASA classification, Age and complications

			Complications, CLavien-Dindo Grade			
		**Total**	**No**	**1-3a**	**3b+**	**p**
ASA1	≤65	174	159 (91%)	14 (8%)	1 (1%)	0.128
	>65	22	18 (82%)	3 (14%)	1 (5%)	
ASA2	≤65	232	207 (89%)	21 (9%)	4 (2%)	0.349
	>65	151	128 (85%)	17 (11%)	6 (4%)	
ASA3	≤65	68	55 (81%)	9 (13%)	4 (6%)	0.176
	>65	88	63 (72%)	13 (15%)	12 (14%)	

ASA: American Society of Anesthesiologists

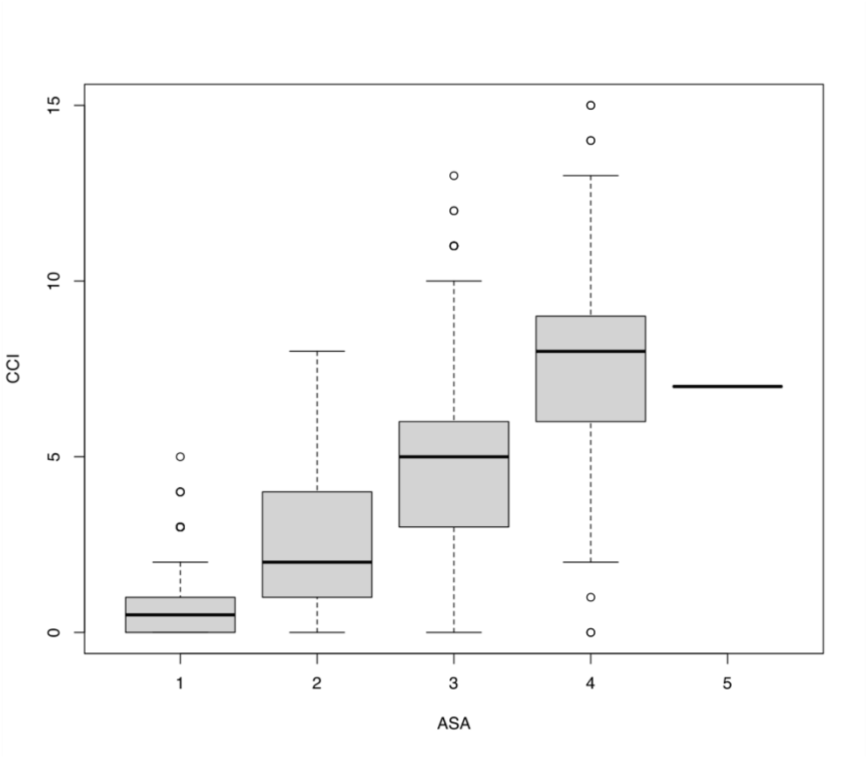

Supplementary Figure 1: Boxplot of CCI score by ASA-classification. Only one patient was ASA5 and had a CCI score of 7
ASA: American Society of Anesthesiologists
CCI: Charlson Comorbidity Index

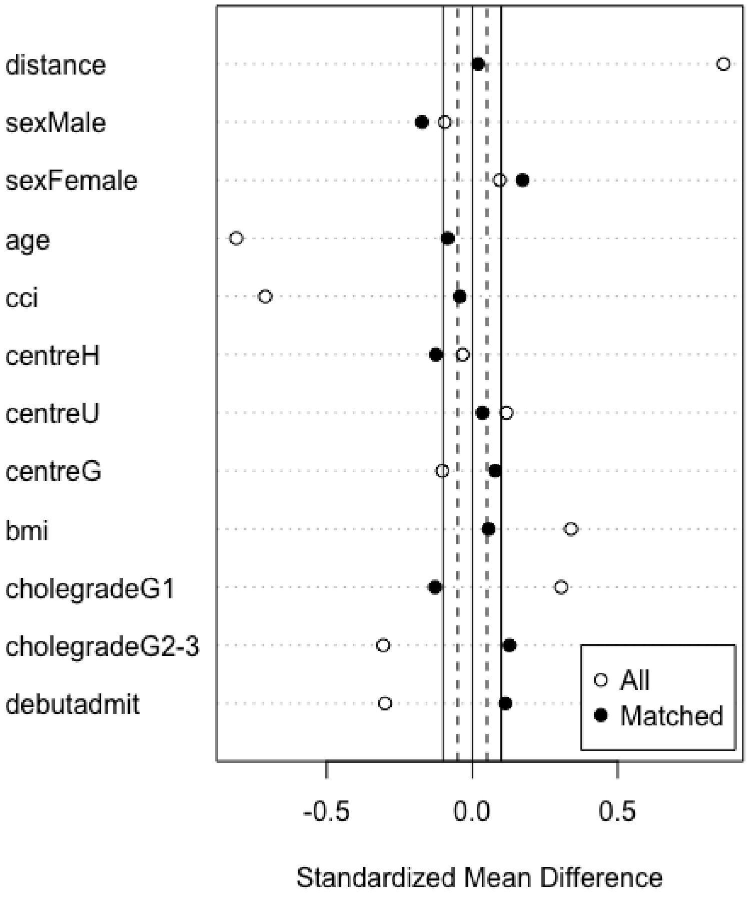

Supplementary Figure 2: Distance between unmatched and matched ASA3 patients.
